# Structure and evolution of the plant cation diffusion facilitator family of ion transporters

**DOI:** 10.1186/1471-2148-11-76

**Published:** 2011-03-24

**Authors:** Jeffery L Gustin, Michael J Zanis, David E Salt

**Affiliations:** 1 Department of Horticulture and Landscape Architecture, Purdue University, 625 Agricultural Mall Drive, West Lafayette, IN 47907-2010, USA; 2 Department of Botany and Plant Pathology, Purdue University, 915 West State Street, West Lafayette, IN 47907-2054, USA; 3 Horticultural Sciences Department, University of Florida, 1117 Fifield Hall, Gainesville, FL 32611-0690, USA

## Abstract

**Background:**

Members of the cation diffusion facilitator (CDF) family are integral membrane divalent cation transporters that transport metal ions out of the cytoplasm either into the extracellular space or into internal compartments such as the vacuole. The spectrum of cations known to be transported by proteins of the CDF family include Zn, Fe, Co, Cd, and Mn. Members of this family have been identified in prokaryotes, eukaryotes, and archaea, and in sequenced plant genomes. CDF families range in size from nine members in *Selaginella moellendorffii *to 19 members in *Populus trichocarpa*. Phylogenetic analysis suggests that the CDF family has expanded within plants, but a definitive plant CDF family phylogeny has not been constructed.

**Results:**

Representative CDF members were annotated from diverse genomes across the Viridiplantae and Rhodophyta lineages and used to identify phylogenetic relationships within the CDF family. Bayesian phylogenetic analysis of CDF amino acid sequence data supports organizing land plant CDF family sequences into 7 groups. The origin of the 7 groups predates the emergence of land plants. Among these, 5 of the 7 groups are likely to have originated at the base of the tree of life, and 2 of 7 groups appear to be derived from a duplication event prior to or coincident with land plant evolution. Within land plants, local expansion continues within select groups, while several groups are strictly maintained as one gene copy per genome.

**Conclusions:**

Defining the CDF gene family phylogeny contributes to our understanding of this family in several ways. First, when embarking upon functional studies of the members, defining primary groups improves the predictive power of functional assignment of orthologous/paralogous genes and aids in hypothesis generation. Second, defining groups will allow a group-specific sequence motif to be generated that will help define future CDF family sequences and aid in functional motif identification, which currently is lacking for this family in plants. Third, the plant-specific expansion resulting in Groups 8 and 9 evolved coincident to the early primary radiation of plants onto land, suggesting these families may have been important for early land colonization.

## Background

Members of the cation diffusion facilitator (CDF) family have been shown to be important for maintenance of cation homeostasis in bacteria, yeast, plants, and mammals [For detailed reviews see references [1-5]]. CDF proteins, in general, bind to and efflux such cations as Zn from the cytoplasm through sequestration into internal compartments or through efflux from the cell. This role in modulating cellular cation concentrations has been demonstrated to impact cation accumulation, cation tolerance, signal transduction cascades, oxidative stress resistance, and protein turnover [6-8].

Several research groups have analyzed the phylogenetic relationships of CDFs and found that this is an ancient gene family that pre-dates the origin of eukaryotes, as reflected in the grouping of sequences from diverse organisms within several branches of constructed phylogenetic trees. Plant CDF members, including 12 members from the sequenced genome of *Arabidopsis thaliana *have been grouped into three or four lineages [2,9,10]. However, these analyses were limited by sequence availability due to the lack of sequence genomes and available cDNA libraries, which resulted in incomplete or weakly supported hypotheses about CDF family phylogeny within plants.

Montanini, *et al. *(2007) conducted global phylogenetic analysis on 273 CDFs from prokaryotes, eukaryotes, and archaea [11]. Based on a maximum parsimony analysis, variation across the gene family could be partitioned into three major groups, designated Zn-CDFs, Zn/Fe-CDFs, and Mn-CDFs based on the hypothesized or confirmed transported substrate of one or more group members. For example, the Mn-CDF group containes 59 sequences and, within this group, the plant members MTP8 and MTP11 have been characterized as Mn transporters. Using vastly expanded sequence information and substrate-defined groups, an updated CDF signature sequence was derived as well as group-specific signature sequences. The conserved residues comprising these signature sequences were the target of amino acid substitution, many of which were found to be critical residues for a fully functional protein. Recently, Migeon *et al. *(2010) expanded this analysis by incorporating CDF sequences from additional plant genomes with emphasis on phylogenetic and molecular characterization of metal transporters in *Populus trichocarpa *[12]. This analysis confirmed partitioning the sequences into three major functional groups. Grouping the sequences by predicted substrate specificity provides a useful hypothesis-generation tool for uncharacterized proteins within these broad groupings. However, higher resolution analysis of plant-specific CDF sequences is likely to reveal informative relationships within the linage of land plants.

With the generation of full genome sequences for multiple eukaryotic organisms, a wealth of information is available from which to generate detailed phylogenomic relationships of gene families within and between organisms. As genome sequences become available for more species, this "genomic" method of phylogenetic analysis should enable robust estimation of orthology and paralogy among related genes. This high level resolution of familial evolution provides a powerful analytical tool from which to synthesize hypothesis about, among other things, the function of gene family members [13]. The precision in functionally annotating an uncharacterized sequence based on sequence similarity to a characterized protein should increase if a detailed estimation of family phylogeny is known [14]. Once a sufficiently detailed map of the gene family structure and evolution are constructed, a more global understanding of the adaptive significance of the family dynamics through the course of evolution may become clearer and lead to testable hypotheses about the roles members play in organismal evolution.

Genome sequencing of a red alga, *Cyanidioschyzon merolae*, green algae, *Ostreococcus tauri*, *Ostreococcus lucimarinus*, and *Clammydomonas reinhardii*, basal nonvascular and vascular land plants, *Physcomitrella patens* (*P. patens*) and *Selaginella moellendorffii*, and representatives of angiosperm lineages have been completed [15-25]. *C. merolae* is a non-motile unicellular red alga that lives in extreme environmental conditions, such as sulfate-rich hot springs and is estimated to have diverged from the lineage leading to true plant (viridiplantae) approximately 1.5 billion years ago [26]. *Ostreococcus *species are the smallest known eukaryotic organisms and belong to the *Prasinophyceae*, an early diverging class in the lineage of the green algae [27-29]. The algal model, *C. reinhardii*, is estimated to have shared a common ancestor with such species as *A. thaliana* 1.1 billion years ago [30]. *P. patens* and *S. moellendorffii* represent early land plant lineages of *Bryopsida *and *Lycopsid*, respectfully, which are estimated to have diverged from seed-bearing plants (Spermatophytes) approximately 480 million years ago (mya) and 400 mya, respectively [31-35]. Within the more recent lineages of flowering plants (angiosperms), several genomes have been sequenced, including the monocotyledonous genomes of *Oryza sativa* (*O. sativa*) and *Sorghum bicolor* (*S. bicolor*), and the eudicotyledonous genomes of *A. thaliana*, *P. trichocarpa*, and *Medicago truncatula*[15-19,22]. The monocot lineage is predicted to have diverged from other angiosperms approximately 200 mya, and within eudicots, the *A. thaliana* and *P. trichocarpa* lineages are predicted to have diverged in the Eurosid clade approximately 120 mya [35-38]. Collectively, the genomes of the six land plants contain information that allow for comparison of genome evolution throughout the approximately 450 million year history of land plants and inclusion of the genomes of red and green algae enables extension to 1.5 billion years of plant evolution.

In this study we conduct a detailed phylogenic analysis of plant CDF family members to lay out a framework from which more informed hypotheses can be generated regarding the function of CDF proteins in plants.

## Results and Discussion

### Plant CDF family member sequences

Scanning the genomes of the taxonomically diverse set of organisms outlined in the introduction for CDF sequences identified or confirmed the following number of sequences: *O. lucimarinus (1)*, *O. tauri (2)*, *C. merolae (3)*, *C. reinhardii (5)*, *P. patens (11)*, *S. moellendorffii (9)*, *O. sativa (10)*, *S. bicolor *(9), *P. trichocarpa (21), and A. thaliana (12) *(Additional File 1). The number of CDF sequences identified from *C. reinhardii, C. merolae*, *S. moellendorffii*, *P. patens*, *S. bicolor*, and *A. thaliana*, genomes agree with previous published studies [2,11,12,39], however, the gene models may not be the same. The number of *P. trichocarpa *CDFs was expanded to 21 from the previous estimate of 19 [12] (Additional File 1). The expanded set includes a predicted pseudogene PtMTP8.4 and previously unidentified PtMTP10.4. The number of CDF sequences in the *O. sativa *genome was expanded from 8 to 10 due to the inclusion of previously unidentified members OsMTP7 and OsMTP8.

### Plant CDF Family Structure

**Figure 1:**
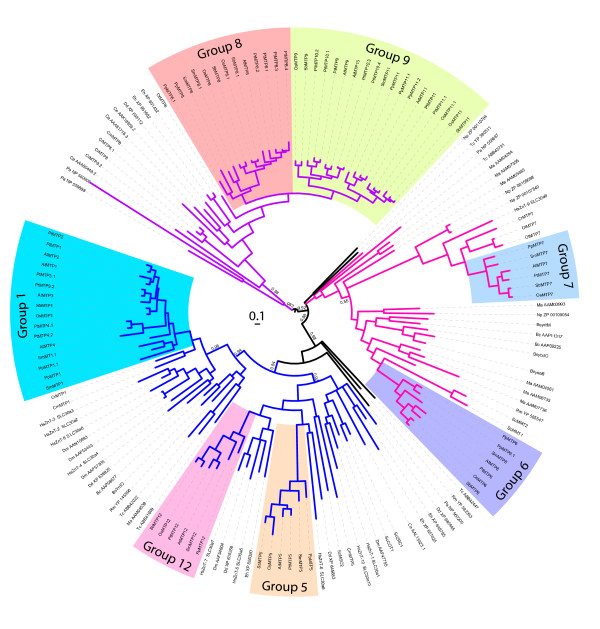
**The CDF superfamily phylogenetic relationships of 151 sequences from diverse taxa were estimated using Bayesian model (MrBayes) and rooted at the calculated midpoint of the two most distant taxa**. Colored branches indicate three primary functional groups of the CDF superfamily defined in [11] and shaded blocks indicate plant-specific groups.

Phylogenetic analysis of the CDF superfamily, including genomes from 2 archaea, 4 bacteria, 2 protozoa, 1 fungi, 1 red alga, 3 green algae, 5 land plants, 1 nematode, and 1 mammal can be grouped into three primary clades, as indicated by the colored branches (lines) in Figure 1. These three primary clades are consistent with the previously defined Zn-CDF, Fe/Zn-CDF, and Mn-CDF groups based on functional evidence of resident members [11], with one exception. While previous analysis of the branch containing HsZNT9 and AtMTP7 had this branch of sequences as an orphaned, ungrouped branch, in this analysis it is included with the Fe/Zn-CDF group, Therefore, this analysis suggests that these sequences be included into the Fe/Zn-CDF group.

CDF family members from Viridiplantae and Rhodophyta genomes were used to estimate the CDF family phylogeny in land plants. The CDF sequences form 7 groups (1, 5, 6, 7, 8, 9, and 12). Groups were defined as lineages originated prior to or at the time of land plant evolution (Figure 1), and group nomenclature was assigned based on annotated CDF sequences from *A. thaliana*. Nomenclature for genes with prior annotations were kept [11,12]. At least one sequence from all six land plant genomes included in this study was maintained in each of the seven groups. CDF members from algae *C. reinhardii*, *C. merolae*, *O. lucimarinus*, and *O. tauri*, are present within 4 of the 7 groups (Figure 1). Maintenance and in some cases expansion of these genes suggests that the CDF members from each group play important roles in plants.

### Group 1

**Figure 2:**
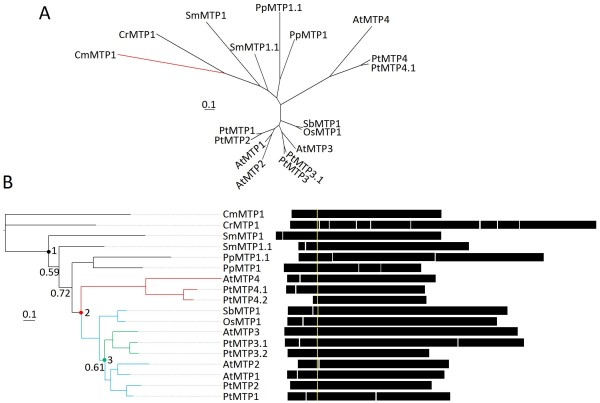
**Group 1 Bayesian-inferred phylogenetic relationships shown as unrooted (A), and rooted (B) trees**. The root for (B) is CmMTP1 (red branch). Branches with posterior probability values less than 0.8 are labeled. A value with an asterisk indicates alternative model sensitivity (see Methods). Exon structure for each gene is displayed. The yellow line indicates predicted position of first residue of the cation efflux domain and models were aligned by this position.

Group 1 originated prior to the evolution of the red alga *C. merolae*, and is maintained in diverse land plant genomes. Group I sequences are found in the both red and green algae, CmMTP1 and CrMTP1, respectively (Figure 2A) [39]. The genomes of *Ostreococcus *do not contain a sequence from Group 1 indicating that this CDF member has been lost in these species. Both *S. moellendorffii *and *P. patens *genomes contain two Group 1 sequences (SmMTP1, SmMTP1.1 and PpMTP1, PpMTP1.1, respectively). The *P. patens *duplication is predicted to have occurred after the mosses diverged from other vascular plant lineages (Figure 2B). However, placement of the two *S. moellendorffii *sequences supports an ancestral duplication event prior to that divergence (Figure 2B, numeral "1") with subsequent propagation of one of the two genes. While the branch support for this model was relatively weak, this topology was consistently supported by both Bayesian and maximum likelihood methods of phylogenetic inference using multiple substitution models. A second duplication event resulted in the formation of lineages containing MTP4 sequences and MTP1/2/3 sequences (Figure 2B, numeral "2"). This analysis supports the evidence that the origin of this duplication occurred prior to the monocot/eudicot divergence due to the presence of monocot and eudicot sequences within the MTP1/2/3 clade. The *S. bicolor *and *O. sativa *genomes lack MTP4 sequences, suggesting that the monocot lineage may have lost this gene. A third duplication event occurring after the monocot/eudicot divergence produced a lineage containing MTP3 sequences and a lineage containing MTP1/2 sequences (Figure 2B, numeral "3"). More recent duplication events within *P. trichocarpa *and *A. thaliana *have generated numerous inparalogs reflecting the genome duplication events that occurred after the divergence of these plants. The *P. trichocarpa *genome contains paralogs for all genes in Group 1, which could reflect the observation that the genome of *P. trichocarpa *is evolving at a six-time slower rate than that of the *A. thaliana *genome, and so might be expected to have a slower rate of loss of duplicated genes [22]. Therefore, Group 1 paralogous genes in *P. trichocarpa *may be highly redundant.

CrMTP1 contains multiple introns and the *P. patens *sequences, PpMTP1 and PpMTP1.1, contain one and two introns, respectively (Figure 2B). The remaining sequences primarily contain only one 5' intron, with a few exceptions. Through searches of public databases, transcript support has been identified for each of the Group 1 members, except for *PtMTP2 *and *PpMTP1.1*. Missing transcript data from *P. trichocarpa *and *P. patens *may be due to incomplete transcript catalogues of these plants. The transcriptional evidence suggests that the genes of Group 1 are largely expressed in a variety of plants and algae, providing further evidence of this group's general importance in plants.

Phylogenetic analysis indicates that the MTP1/2 sequences and MTP3 sequences share a common ancestor some time after the monocot/eudicot divergence (Figure 2B). At the time of duplication, MTP1/2 and MTP3 most likely shared identical redundant function in that ancestor. The fate of the duplicated genes could take several different paths, including elimination, neofunctionalization, subfuctionalization, or even full/partial redundancy [40]. The *AtMTP1 *and *AtMTP3 *DNA sequences share 67.7% sequence identity, and the proteins have similar predicted secondary structure with six transmembrane domains, cytoplasmically facing N-terminal and C-terminal ends, and a histidine-rich region [2,41,42]. Both proteins have been localized to the tonoplast membrane in yeast and plants, and both proteins have been shown to affect Zn and possibly Co tolerance and accumulation in yeast [41,43-46]. However, the spatial, temporal, and responsive transcriptional regulation of each gene suggests that these proteins have different roles in plant Zn homeostasis. Evidence from an *A. thaliana *relative, *Brassica juncea*, suggests that *BjMTP1 *is expressed in secondary xylem parenchyma cells of the root while *AtMTP3 *is expressed in root epidermal and cortical cells [41,47]. Also, *AtMTP1 *and *BjMTP1 *transcription is not regulated by Zn, while *AtMTP3 *is activated by elevated Zn influx [41,43,47]. Therefore, when *MTP3 *is expressed in conditions of high Zn or low Fe, accumulation of MTP3 and MTP1 could provide a continuous sequestration path in epidermal/cortical cell layers and xylem parenchyma cells limiting Zn translocation to the shoot [5,41]. Spatial expression patterns of *MTP1 *and *MTP3 *are also different in vegetative and inflorescent shoot tissues [41,43,47]. So, while the protein sequence, structure, location, and substrate(s) are very similar, the expression patterns between *AtMTP1 *and *AtMTP3 *are unique. Therefore maintenance in the genome of the originally duplicated genes may be attributed to neofunctionalization/subfunctionalization via changes in expression patterns of the gene.

Additionally, the genome of *A. thaliana *maintains a more recent (<120 mya) duplication event yielding sequences AtMTP1 and AtMTP2 (Figure 2B). Comparing their gene expression metaprofiles across a database of microarrays suggests that they are not coexpressed (R^2 ^= 0.001) [48], which suggests that these paralogs are not redundant.

### Groups 8 and 9

**Figure 3:**
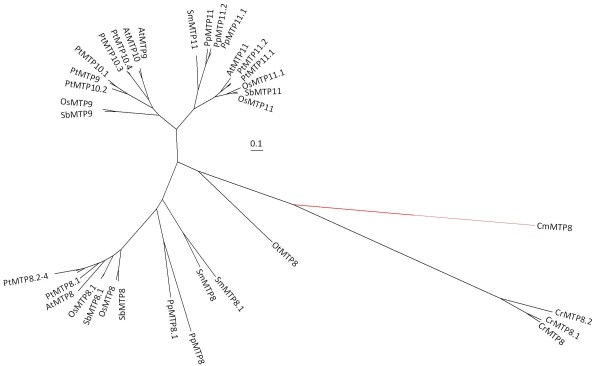
**Bayesian-inferred phylogenetic relationships of Groups 8 and 9 sequences**. All posterior probability values less than 0.8 are indicated. A value with an asterisk indicates alternative model sensitivity (see Methods).

**Figure 4:**
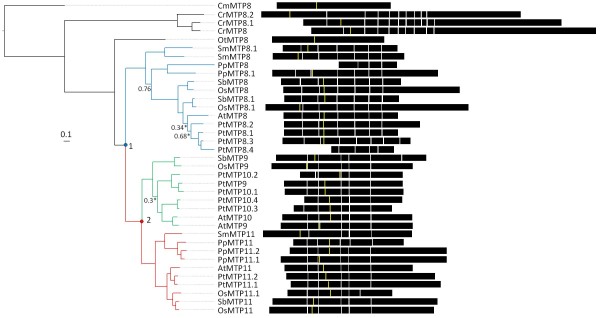
**Bayesian-inferred phylogenetic relationships shown as rooted trees for Groups 8 (blue) and 9 (red) sequences**. The root for (B) is CmMTP8 (red branch shown in Figure 3). All posterior probability values less than 0.8 are indicated. A value with an asterisk indicates alternative model sensitivity (see Methods). Exon structure for each gene is displayed. The yellow line indicates predicted position of first residue of the Cation Efflux domain and models are aligned by exon structure. PpMTP8 and PtMTP8.4 do not contain a predicted Cation Efflux domain.

This clade of the CDF superfamily tree contains CDF members from genomes of both red and green algae (CmMTP8, CrMTP8, CrMTP8.1, CrMTP8.2, and OtMTP8), and the presence of other prokaryote and eukaryote CDF sequences supports the ancient origin of this clade (Figures 1 and 3A). Representing the Viridiplantae sequences of this clade as rooted by the red algae sequence, CmMTP8, indicates that a duplication event (Figure 4 numeral "1") within this branch of the CDF family occurred to produce two distinct groups, Group 8 and Group 9 (Figure 4). The duplication event appears to have occurred prior to or coincident with early land plants due to the presence of *P. patens *and *S. moellendorffii *sequences in both groups. The two monocot genomes each contain two Group 8 sequences (MTP8 and MTP8.1) and phylogenetic relationships between these sequences suggest that they are a product of a duplication event that occurred in a common ancestor of *O. sativa *and *S. bicolor*. Indeed, the MTP8 and MTP8.1 sequences from rice and sorghum fall within syntenous blocks between their respective genomes, confirming duplication within the pre-grass ancestor [49]. Group 9 shows evidence of a duplication event prior to the moncot/eudicot split (Figure 4 numeral "2"). This duplication event produced two Group 9 lineages in higher plants, both of which are maintained, and in some cases expanded, in representative genomes. In the *P. trichocarpa *genome, Groups 8 and 9 contain 11 CDF members (4 sequences in Group 8 and 7 sequences in Group 9). Amplification of these groups in *P. trichocarpa *is primarily the result of tandem gene replication of three of the Group 8 sequences (PtMTP8.2 to 8.4) and 4 of the Group 9 sequences (PtMTP10.1 to 10.4). Members of Groups 8 and 9 have been functionally characterized as Mn transporters. The first member of these groups to be cloned was *ShMTP8 *(originally *ShMTP1*) from the Mn hyperaccumulating legume, *Stylosanthes hamata*. The clone was identified from a screen for cDNAs that enhanced Mn tolerance in yeast [50]. cDNA sequences for three other Group 8 and 9 members were also identified from the screen. Fluorescent tagging of ShMTP8 suggested that the protein functions at the tonoplast where it was predicted to be involved in Mn sequestration into the vacuole. Group 9 sequences, AtMTP11, PtMTP11.1, and PtMTP11.2 have also been characterized as Mn transporters. However, these proteins reside not within the vacuole, but within a punctate endomembrane compartment consistent with either trans-Golgi or prevacuolar organelles [51,52]. Deletion of the *AtMTP11 *gene product increased accumulation of Mn in leaves of plants grown *in vitro *or hydroponically [51,52]. Deletion or reduction of *AtMTP11 *transcripts makes the mutant plant sensitive to elevated Mn, whereas ectopic over expression of *AtMTP11 *increases resistance to elevated Mn. It is clear that CDFs from Group 8 and 9 are important for Mn homeostasis and the early bifurcation and subsequent expansion of these gene families implies an adaptively significant role for Mn homeostasis in plants.

The intron-exon boundaries largely support the evolutionary relationships of these sequences. In Group 8, two gene models, *PtMTP8.4 *and *PpMTP8 *(Figure 4), do not conform to a seven-exon gene structure. These loci have no associated ESTs, and when compared to their respective Group 8 sequences, both loci have large truncations of 5' regions that eliminate large portions of the cation efflux domains. This suggests that these loci are pseudogenes. Group 9 angiosperm sequences have very similar gene models (Figure 4). The exon boundaries of the *S. moellendorffii *and *P. patens *sequence deviate slightly from those defined in the angiosperms, but a clear 6 exon pattern is evident for most Group 9 sequences.

### Groups 5 and 12

**Figure 5:**
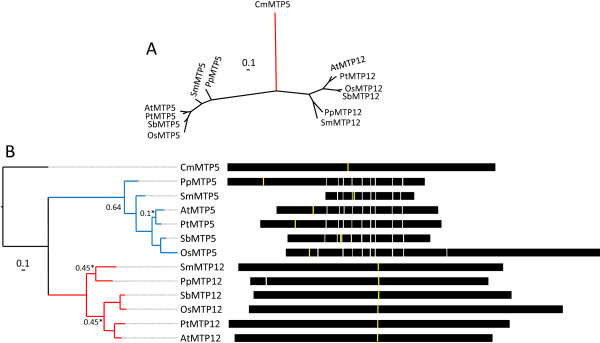
**Bayesian-inferred phylogenetic relationships shown as unrooted (A), and rooted (B) trees for Groups 5 (blue) and 12 (red) sequences**. The root for (B) is CmMTP5 (red branch in the unrooted tree). All posterior probability values less than 0.8 are indicated. A value with an asterisk indicates alternative model sensitivity (see Methods). Exon structure for each gene is displayed. The yellow line indicates predicted position of first residue of transmembrane 8 for Group 12 and transmembrane 1 for Group 5. Models were aligned by this position.

Group 5 and Group 12 lineages derive from a common ancestor prior to the origin of land plants and within each lineage are sequences from prokaryotes and eukaryotes, thus showing that each group is of ancient origin (Figure 1). Unlike Groups 1, 8, and 9, green algal sequences are absent from these groups. However, a CDF sequence from red algae, CmMTP5, which is distantly related to either Group 5 or Group 12, falls within the larger grouping of these sequences in the superfamily tree (Figure 1), so this sequence was used as the root to estimate the phylogenetic relationships within these groups (Figure 5A &5B). Each group contains only one sequence from each of the included plant genomes, implying strict maintenance of a single gene copy within genomes, unlike in Groups 1, 8, and 9. Group 12 sequences are maintained as large single-exon genes, while the gene structure of Group 5 sequences contain numerous intons with reasonably well maintained exon structure (Figure 5B). Group 5 sequences are also relatively variable in size, and all but SmMTP5 have associated cDNA or EST support. Only two members of Group 12 are supported by ESTs, PtMTP12 and PpMTP12. The sequences that make up Group 12 are of note because the average sequence of these members is approximately twice the length of a typical CDF sequence. The cation efflux domain starts at the center and extends toward the 3' end of the gene, while the 5' half of the gene (approximately 1200 bp) does not show clear homology to other genes or to known functional domains.

Function evidence for the role of Group 5 or Group 12 genes in plants is limited. The only functional data for these groups comes from the high throughput ionomic phenotyping database in which diverse plant accessions are screened for ionomic profiles [53]. Among the many mutant lines screened by this group was an EMS induced mutation of *AtMTP5*. The ionomic profile of this mutant shows repeatable alterations in multiple ions in the mutant leaves including reduced levels of Mo, Mn, and Mg and increased levels of K and Zn. These data suggest that AtMTP5 has a role in regulating ion concentrations in *A. thaliana *under normal conditions.

### Groups 6 and 7

**Figure 6:**
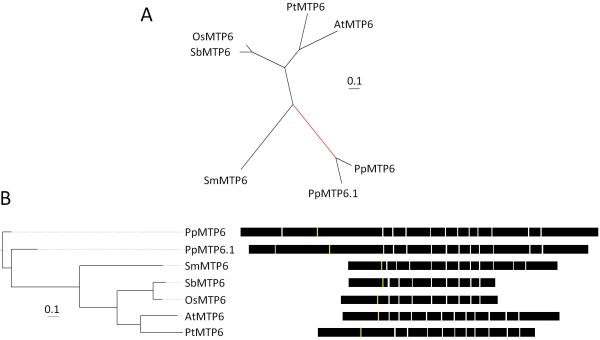
**Bayesian-inferred phylogenetic relationships shown as unrooted (A), and rooted (B) trees for Group 6 sequences**. The root for (B) is the branch leading to PpMTP6 and PpMTP6.1 (red branch). All posterior probability values less than 0.8 are indicated. A value with an asterisk indicates alternative model sensitivity (see methods and materials). Exon structure for each gene is displayed. The yellow line indicates predicted position of first residue of the cation efflux domain and models are aligned by exon structure.

**Figure 7:**
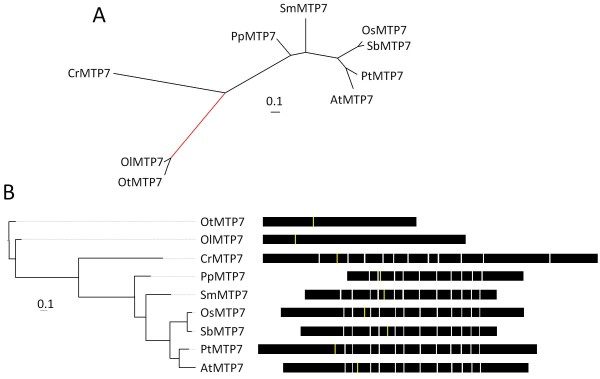
**Bayesian-inferred phylogenetic relationships shown as unrooted (A) and rooted (B) trees for Group 7 sequences**. The root for (B) is the branch leading to OtMTP7 and OlMTP7 (red branch in the unrooted tree). All posterior probability values less than 0.8 are indicated. A value with an asterisk indicates alternative model sensitivity (see Methods). Exon structure for each gene is displayed. The yellow line indicates predicted position of the first residue of the Cation Efflux domain and models are aligned by exon structure.

Group 6 and Group 7 plant sequences each belong to lineages that radiate from the base of the unrooted CDF superfamily tree and each lineage includes other CDF sequences from diverse organisms demonstrating that these groups are of ancient origin (Figure 1). Rooting Group 6 with the branches leading to the *P. patens *sequences, PpMTP6 and PpMTP6.1, and rooting Group 7 with the branch leading to the *Ostreococcus *sequences, OlMTP7 and OtMTP7, produces the cladistic relationship among the sequences (Figures 6B and 7B, respectively). Similar to Groups 5 and 12, plants have maintained only one copy of Group 6 and 7 sequences in their genomes, and 11 of the 14 sequences from these groups are supported by ESTs or cDNAs. The genomes of the green and red algae representatives included in this analysis do not contain Group 6 sequences, suggesting that the Group 6 members in algae have been lost (Figure 6A) [39].

The Group 6 members are the only plant CDF sequences to fall into the Zn/Fe-CDF group, although no studies have been conducted on Group 6 plant family members to confirm this substrate specificity [11]. The only functional data for these groups comes from ionomic phenotyping [53]. Profiling of an *A. thaliana *line with a homozygous T-DNA insertion into the coding region of *AtMTP6 *shows consistent diverse alterations in the ionome with reduced levels of Mg, Mo, and Ca and increased levels of Na, K, Mn, and Cd. The altered ion profile of the *mtp6 *mutant leaves suggests that Group 6 sequences are required for the maintenance of the plant ionome under normal conditions. The Group 7 sequences were not placed into any of the three substrate-specific groups and no functional data are available for members of this group [11].

## Conclusions

Studies in mammals, nematodes, yeast, bacteria, and plants suggest CDF proteins serve important roles in essential cation transport and homeostasis. There is also evidence supporting other, more complex, roles in these organisms, such as involvement in oxidative stress resistance, interactions in signal transduction cascades, and proper functioning of the endoplasmic reticulum. Within plants only four members have been functionally characterized to any degree, and these studies show the importance of each member in essential cation accumulation, partitioning, and tolerance. Using phylogenomic analysis of complete CDF families from genomes of multiple, taxonomically diverse plants and algae, the plant CDF family is organized into seven primary groups that were present in ancestral genomes prior to or coincident with the origin of land plants. Within land plants, gene copy number expansion continues within select groups, while several groups are strictly maintained as one gene copy per genome. Defining these CDF lineages contributes to the study of this family in four ways.

1) Defining within group orthology/paralogy of particular genomes will help highlight potential redundant genes. For example, the *P. trichocarpa *genome has six Group 1 members, however these six sequences are actually three separate recent duplications of members in three different clades within Group 1 (Figure 2). This might predict that the protein products of the recently duplicated genes (i.e., PtMTP3.1 and PtMTP3.2) may have redundant function, but the inparalogs (PtMTP1 and PtMTP3.1) might not be redundant, but rather are subfunctionalized members similar to AtMTP1 and AtMTP3 (see discussion on Group 1, above).

2) Defining the primary groups improves the predictive power of functional assignment of orthologous/paralogous genes and aids in hypothesis generation when embarking upon functional studies of the members. For example, plant sequences from Groups 1, 5, 6, 7, and 12 are likely monophyletic lineages derived within ancestral prokaryotes and largely maintained in extant organisms. This suggests that comparisons with bacterial, archaeal, fungal, and mammalian homologues may be useful. Conversely, Group 8 and 9 lineages most likely result from a duplication of an ancestral Viridiplantae sequence. Therefore, sequences within at least one of these groups might have an altered functional role in plants as compared with the function of coorthologs in other organisms.

3) Defining groups will allow for a group-specific sequence motif to be generated that will help define future CDF family sequences and aid in functional motif and critical residue identification in plants. A CDF family signature sequence was defined that identifies CDF family members with only a 5% false identification rate, but this sequence is quite elaborate [11]. The necessarily complex signature sequence may reflect the constraints inherent in encompassing all CDF family members and includes all variations within a diverse set of organisms. By focusing specifically on plant CDF members, the sequence variability due to host genome diversity will be reduced leading to more accurate identification of group-specific sequence motifs and critical residues important in plant CDF proteins.

4) The plant-specific expansion resulting in Groups 8 and 9 evolved prior to or coincident with the early primary radiation of plants onto land. The primary Siluro-Devonian radiation of terrestrial plants necessitated development of physiological mechanisms that would allow pioneering plants to take advantage of new ecological niches on land. In terms of the CDF family, the expansion from five to seven primary groups prior to or coincident with the divergence of bryophytes from the vascular plant lineage suggests the CDF family expansion provided an adaptive advantage before significant vascular development occurred in early land plants.

## Methods

### Sequence Identification

Protein sequences from *A. thaliana *were obtained from the NCBI database http://www.ncbi.nlm.nih.gov/. Gene models and protein sequences from *O. sativa *ssp. *japonica, P. trichocarpa, S. bicolor*, and *C. reinhardtii *were identified from the Phytozome website http://www.phytozome.net/ using the tBLASTn algorithm with the twelve *A. thaliana *CDF protein sequences [54,55]. The gene model for *OsMTP11.1 *used in this study was obtained from the The Institute for Genomic Research (TIGR) website http://plantta.jcvi.org/ because the Phytozome gene model appears to be incorrect based on multiple sequence alignment with Group 9 sequences. Gene models of CDF family members from S. *moellendorffii *and *P. patens *were identified and annotated from the *S. moellendorffii *genome browser http://genome.jgi-psf.org/Selmo1/Selmo1.home.html and the *P. patens *resources website http://www.cosmoss.org/, respectively, through homology to *A. thaliana *CDF members by tBLASTn algorithm. CDF family members from *O. tauri*, *O. lucimarinus*, and *C. merolae *were identified through homology to *S. moellendorffii *CDF members by tBLASTn searches of their respective genome assemblies located at the Department Of Energy Joint Genome Institute (DOE JGI) http://www.jgi.doe.gov/ and the *Cyanidioschyzon merolae *Genome Project http://merolae.biol.s.u-tokyo.ac.jp/. Sequences from the genomes of *M. acetivorans *C2A, *B. cereus *ATCC 14579, *N. punctiforme *PCC 73102, *E. histolytica *HM-1:IMSS, *D. melanogaster*, *C. elegans*, *S. cerevisiae*, and *H. sapien *used for the CDF superfamily analysis (Additional File 2) were retrieved from the GenBank database using the accession numbers provide by [11]. The CDF family members from *P. aerophilum *str. IM2, *R. metallidurans *CH34, *T. crunogena *XCL-2, and *D. discoideum *AX4 were identified from their respective sequenced genomes by tBLASTn using bacterial CDF sequences.

### CE Sequence Alignment and Phylogenetic analysis

Protein sequences were aligned with ClustalW with using the Gonnet series weight matrix and default parameters http://workbench.sdsc.edu[56]. Phylogenetic analysis was conducted by MrBayes, Bayesian inference of phylogeney, http://mrbayes.csit.fsu.edu/index.php with the amino acid model set to pr = mixed and lset rates = gamma [57,58]. Two independent chains of Markov Chain Monte Carlo (MCMC) analysis were allowed to run until the standard deviation of the split frequencies was stable (ngen = 100,000-200,000)(SumT PRSF = ~1.0). The output file was read into the Interactive Tree of Life (iTOL) tool http://itol.embl.de/ for visualization and editing [59]. Node probability values (posterior probability values) below 0.8 are shown in the figures. To test the accuracy of the tree topologies generated by the ClustalW alignments and Bayesian analysis, each group was also subjected to an alternative alignment by Muscle [60] and mafft [61] and alternative phylogenetic analysis by maximum likelihood (ML) using phyML [62] with LG and JTT substitution models and rate heterogeneity. In cases where the alternative algorithms indicated weaker branch support than the ClustalW/MrBayes predictions, the probability values of the alternative algorithms are included in the figures as posterior probability values with an asterisk. In large part, the alternative topologies agreed with those produced using ClustalW and MrBayes. One exception was Group 1. The topology of this group was sensitive to the method of alignment. Group 1 tree topologies generated by the Muscle and mafft alignments were consistent and contradicted topologies predicted by ClustalW alignments at several branches. However, the group was not sensitive to phylogenetic model selection as both MrBayes and phyML generated consistent topologies for a given alignment irrespective of the substitution model. Due to the consistent phylogenies produced by the Muscle and mafft alignments, the Muscle alignment was used for the phylogenetic analysis in Figure 2.

### Nomenclature

If annotations were lacking for plant CDF family members, annotations were given in accordance with the *A. thaliana *CDF family in most cases (Additional File 1) with the nomenclature model [1^st ^letter of genus name][1^st ^letter of species name]["MTP"][group number], for example, AtMTP1. In cases where one group contained multiple sequences from one plant, paralogous sequences are denoted with [group name][.n] where n is a number (1,2,3) that reflects sister lineage in cases where such predictions can be made. Established gene names were kept for *A. thaliana *and *P. tricocarpa *CDFs to maintain continuity between published studies. Changes to established annotations were recommended for *C. reinhardtii *to reflect each sequence's position in the phylogenetic tree. Additional File 1 lists the given names and the accession numbers used to identify the annotations in the given genome.

## Abbreviations

Abbreviations for Figures 1, 2, 3, 4, 5 and 6 and Additional files 1, 2 are as follows: At: *Arabidopsis thaliana *(thale cress); Os: *Oryza sativa *(rice); Sb: *Sorghum bicolor *(sorghum), Pt: *Populus trichocarpa *(black cottonwood); Cr: *C. reinhardtii *(green algae); Sm: *Selaginella moellendorffii *(spike moss), Pt: *Physcomitrella patens *(moss); Ot: *Ostreococcus tauri *(phytoplankton); Ol: *Ostreococus lucimarinus *(phytoplankton); Cm: *Cyanidioschyzon merolae *(red algal). Additional abbreviations for Figure 1 are as follows: Pa: *Pyrobaculum aerophilum *str. IM2; Ma. *Methanosarcina acetivorans *C2A; Bc: *Bacillus cereus *ATCC 14579; Rm: *Ralstonia metallidurans *CH34; Tc: *Thiomicrospira crunogena *XCL-2; Np: *Nostoc punctiforme *PCC 73102; Dd: *Dictyostelium discoideum *AX4 (slime mold); Eh: *Entamoeba histolytica *HM-1:IMSS (amoeba), Dm: *Drosophila melanogaster *(Fruit Fly); Ce: *Caenorhabditis elegans *(nematode); Hs: *Homo sapien *(human); Sc: *Sacchromyces cerevisiae*, (baker's yeast).

## Authors' contributions

All authors have read and approved the final manuscript. JLG conducted the search and annotation of the CDF sequences, preformed the phylogenetic analyses, wrote the manuscript, and prepared the figures with guidance from DES and MJZ.

## Supplementary Material

Additional file 1 **CDF members from genomes of photosynthetic eukaryotes used in this study**. Definitions/Accessions and associated databases where sequence annotations are deposited are given. Click here for file

Additional file 2 **CDF members from diverse genomes used to create the CDF superfamily phylogenetic tree**. Organism list is abbreviated and updated from Montanini *et al. *(2007) to reflect sequenced genomes representative of diverse taxonomic sampling. GenBank definitions and accession numbers are given. Click here for file
